# Moral cognition, the missing link between psychotic symptoms and acts of violence: a cross-sectional national forensic cohort study

**DOI:** 10.1186/s12888-019-2372-4

**Published:** 2019-12-19

**Authors:** Ken O’Reilly, Paul O’Connell, Danny O’Sullivan, Aiden Corvin, James Sheerin, Padraic O’Flynn, Gary Donohoe, Hazel McCarthy, Daniela Ambrosh, Muireann O’Donnell, Aisling Ryan, Harry G. Kennedy

**Affiliations:** 1 National Forensic Mental Health Service, Central Mental Hospital, Dundrum, Dublin, Ireland; 2 Department of Psychiatry, Trinity College, Dublin, Ireland; 3 Department of Psychology, University of Regensburg, Regensburg, Germany; 4 Department of Psychology, National University of Ireland Galway, Galway, Ireland

**Keywords:** Violence, Homicide, Schizophrenia, Moral cognition, Delusions, Hallucinations, Violence risk assessment, Neurocognition, Forensic psychiatry, Moral foundations theory

## Abstract

**Background:**

People with schizophrenia are ten times more likely to commit homicide than a member of the general population. The relationship between symptoms of schizophrenia and acts of violence is unclear. There has also been limited research on what determines the seriousness and form of violence, such as reactive or instrumental violence. Moral cognition may play a paradoxical role in acts of violence for people with schizophrenia. Thoughts which have moral content arising from psychotic symptoms may be a cause of serious violence.

**Method:**

We investigated if psychotic symptoms and moral cognitions at the time of a violent act were associated with acts of violence using a cross-sectional national forensic cohort (*n* = 55). We examined whether moral cognitions were associated with violence when controlling for neurocognition and violence proneness. We explored the association between all psychotic symptoms present at the time of the violent act, psychotic symptoms judged relevant to the violent act and moral cognitions present at that time. Using mediation analysis, we examined whether moral cognitions were the missing link between symptoms and the relevance of symptoms for violence. We also investigated if specific moral cognitions mediated the relationship between specific psychotic symptoms, the seriousness of violence (including homicide), and the form of violence.

**Results:**

Psychotic symptoms generally were not associated with the seriousness or form of violence. However, specific moral cognitions were associated with the seriousness and form of violence even when controlling for neurocognition and violence proneness. Specific moral cognitions were associated with specific psychotic symptoms present and relevant to violence. Moral cognitions mediated the relationship between the presence of specific psychotic symptoms and their relevance for violence, homicide, seriousness of violence, and the form of violence.

**Conclusions:**

Moral cognitions including the need to reduce suffering, responding to an act of injustice or betrayal, the desire to comply with authority, or the wish to punish impure or disgusting behaviour, may be a key mediator explaining the relationship between psychotic symptoms and acts of violence. Our findings may have important implications for risk assessment, treatment and violence prevention.

## Background

Although most people with psychotic disorders such as schizophrenia or schizoaffective disorder are not violent, there is an association between psychotic disorders and violence, and with homicide in particular [1, 2]. Patients with schizophrenia are ten times more likely to commit homicide than a member of the general population [1]. However, the proportion of homicides in society attributable to schizophrenia is small and consistently falls below 10% [3, 4]. Also patients with schizophrenia are themselves fourteen times more likely to become victims of violence in the community, compared with being arrested as a perpetrator [5]. Notwithstanding these findings, understanding the relationship between psychosis and violence is important because it may help reduce violence within this population, save lives, and prevent patients becoming stigmatized by an act of violence arising from their mental disorder.

Attempts to explain rare but serious acts of violence by people with mental disorders have a long history. The M’Naughten rules are the paradigmatic example of a rational attempt to explain the relationship between the symptoms of these disorders and violence [6]. An essential element of this legal defence is that a person found ‘not guilty by reason of insanity’ (NGRI) had an ‘innocent intent’. For a verdict of insanity, it must be demonstrated that at the time of committing the act an individual was “*labouring under such a defect of reason, from a disease of the mind, as not to know the nature and quality of the act he (she) was doing, or, if he did know it, that he did not know that he was doing wrong”* [6]*.* Both loss of contact with reality, for example delusions and hallucinations, and confused moral reasoning are accepted as mitigating and by implication explanatory factors. Although nearly two-hundred years old, this defence has proven robust to criticism and is still widely used today [7]. To date within the forensic literature, emphasis has been placed on empirically investigating psychotic symptoms, such as delusions and hallucinations, for understanding acts of violence [8–11]. In contrast, the role played by the form and content of the associated moral reasoning has seldom been investigated [12, 13].

### Empirical investigations of the relationship between delusions, hallucinations, and violence

Empirical investigations of whether delusions and hallucinations are determinants of violence have undergone three iterations. The first iteration explored the epidemiology of psychotic symptoms and violence across community, prison, and forensic hospital samples [14–17]. These epidemiological studies reported a statistical association between psychotic symptoms and violence, such as threat-control override (delusions or hallucinations of imminent threat or external control) but did not clarify causal relationships. Many patients with schizophrenia or schizoaffective disorder will have experienced psychotic symptoms despite having no history of violence, indicating that symptoms are not sufficient for violence to occur. Others often treated within forensic services will have carried out a single but serious act of violence despite a long history of experiencing symptoms [1, 2, 15, 16]. The second iteration took a prospective approach in an attempt to unpack causal relationships. In contrast to the associations observed in cross-sectional or retrospective studies, findings from some prospective studies found neither delusions in general, nor threat control override delusions in particular were associated with a higher risk of violent behaviour [8, 9]. A limitation of these prospective studies is that many patients who were experiencing psychotic symptoms at baseline were asymptomatic at follow up, therefore obscuring causal relationships [9–11]. The third iteration therefore sought to address these limitations and placed special emphasis on whether emotions like anger were the missing link and mediated the relationship between delusions and violence [10, 11, 18, 19]. These studies reported a link between delusions and violence when anger was present, but many patients who experienced angry affect in conjunction with delusions were nonviolent [9–11]. This approach to ‘realist’ evaluation of mechanisms within mental health has wide value [20–22]. Finally, psychotic symptoms are not always the sole motivator for acts of violence amongst patients with mental illnesses [23]. Therefore, uncertainty remains as to whether delusions or hallucinations are determinants of violence, or whether angry affect or other factors are key mediators. The association between psychotic symptoms and acts of violence remains unexplained.

### The relevance of moral cognitions for violent acts

It is surprising that moral cognition has not been investigated as an important mediator given that laws for distinguishing those incapacitated because of mental illness from the criminally culpable emphasize confused moral reasoning [7, 12]. The psychology of moral cognition, namely what a person considers to be moral, is relevant for understanding serious violence because moral cognitions have a distinct set of qualities. Moral cognitions are actionable (i.e. they demand action or compel a person to act), they are universally applied (i.e. they are independent of local customs, rules or laws), and moral infractions are punishable, which may involve violence [24–32]. Moral cognitions may subjectively justify violence to the point of homicide in individuals who are not particularly prone to violence or who have no history of violence. Recent evidence suggests that combatants are willing to die for what they perceive to be moral cognitions, even prioritising them over kin relations [33]. Serious violence like homicide is particularly likely to have a moral component [12, 24, 34]. Whilst the manifest reasons for the majority of homicides feature what criminologists have described as ‘trivial altercations’, the latent reasons concern powerful moral sentiments such as injustice and betrayal [34, 35]. Homicide also differs from many violent acts because of its high detection and clearance rate (i.e. completed police investigation), in addition to being associated with the most severe punishments [36, 37]. It is counterintuitive to think that moral cognition is a cause of homicide, but the qualities of moral cognition help explain why the perpetrators of homicide appear to be acting against their own best interest. Those who commit homicide may be willing to risk conviction and punishment in order to act in accordance with their moral cognitions [25, 26, 30, 31, 32, 37, 38]. Given that moral cognition is relevant for acts of violence amongst different populations [12, 13, 24, 33], and because of the properties of moral cognition itself [24–30], we speculated that moral cognition would play an important role for acts of violence by forensic patients.

### Moral foundations theory

Several different schemes have been developed for categorising and quantifying moral cognition [24, 38, 39]. Moral foundation theory for example, proposes five basic forms of moral cognition which are thought to be universal, innate, and adaptive [39]. The theory finds parallels in other structuralist schemes such as innate language acquisition and universal emotional expression [40, 41]. Haidt’s moral foundation theory categorises moral cognitions into ‘care-harm’, ‘fairness-reciprocity’, ‘in-group-loyalty’, ‘authority-respect’ and ‘purity-sanctity’, all of which can paradoxically lead to violence if prioritised over life [42]. Mercy killings, feuds, crimes of passion, punishments, and honour killings are all associated with specific moral foundations [24, 34, 42, 43]. An individual’s moral dispositions or traits can be quantified using the moral foundations questionnaire (MFQ-30), a measure of one’s endorsement of each of Haidt’s categories of moral cognition [44]. Universal moral foundations may differ in content in various cultures depending on how behaviours within a culture are categorised. While different cultures may regard some actions as unclean and others chaste, all cultures recognise impure acts. Moral cognitions associated with specific moral foundations may be particularly important for determining the seriousness of violence [12], in addition to the form of violence [45].

### Instrumental and reactive violence

Violence occurs in different forms. Reactive violence is primarily emotional, impulsive, and defensive, whereas instrumental violence is primarily predatory, involving goal setting and planning [46]. Both forms of violence are underpinned by different neural systems [47]. The distinction between reactive and instrumental violence may be important for understanding and managing violence carried out by patients with schizophrenia particularly because instrumental violence may be precipitated by less obvious warning signs [47]. The cognitive impairments characteristic of schizophrenia may be a determinant of reactive violence [48, 49], which is executively simple, whilst simultaneously being protective for instrumental violence, which is executively complex [48, 49]. In keeping with these findings, there have been calls for research on violence associated with schizophrenia to pay greater attention to the form of violence to help clarify causal relationships [48–52].

### Research on moral cognition and acts of violence for patients with schizophrenia or schizoaffective disorder

Previously it has been shown that moral cognition is relevant to acts of serious violence by patients with psychotic disorders [12, 13]. Within a national cohort of forensic patients, those who committed homicide scored higher on the Moral Foundations Questionnaire-30 (MFQ-30), compared to those who did not commit homicide but were otherwise violent. Moral cognition also mediated the relationship between neurocognition and homicide [12], suggesting that patients with cognitive impairments were more likely to kill if they held strong moral values. However, the MFQ-30 is a measure of moral dispositions or traits rather than mental states. Moreover, it is possible for a person to experience moral cognitions associated with a particular moral foundation even if they are not particularly elevated on the moral trait e.g. fairness-reciprocity. Separately and independent of our research on the MFQ-30 and homicide, Friedman et al. [13] found that amongst a sample of female patients who had killed their children “Over half (54%) of the mothers killed for “altruistic” reasons; most (85%) of the “altruistic” motivations were psychotic. For example, a common psychotic “altruistic” motive was killing to prevent the child from being tortured, such as by a demon. Consequently, moral cognitions or mental states experienced at the time of a violent act may be particularly relevant for understanding why some patients with schizophrenia act on their delusions, and others do not [12, 14, 24].

### Psychotic symptoms and moral cognitions arising from impaired information processing

Some contemporary cognitive accounts of delusions suggest that they are ‘beliefs’ arising from impaired information processing [53]. According to one formulation, a delusion may be the product of a faulty appraisal of an experience or inability to rationally criticise the experience [53]. Moral cognitions may also arise during information processing. Delusions and hallucinations may frame a situation in a way that triggers moral cognition (where moral cognitions as outlined above may be regarded as universal, actionable and punishable). However, this moral appraisal may or may not be ‘moral’ in the philosophical sense even though the person subjectively believes they are doing the ‘right thing’. For example, if a person believes that children are to be killed, and there is not sufficient time to contact the authorities, they may make the appraisal that they are justified in acting violently to prevent or reduce suffering. Moral cognitions may therefore mediate the relationship between psychotic symptoms and acts of violence.

### A priori association between psychotic symptoms and moral cognitions

For moral cognition to mediate the relationship between delusions, hallucinations and violence, specific moral cognitions must be associated with specific psychotic symptoms. Many delusions and hallucinations relevant to violent acts appear to have a moral component. Factor analysis of delusions and hallucinations produces dimensions [54], which overlap with moral foundations theory. For example, one study involving 660 psychotic patients produced five factors [54]: the first of which included grandiose delusions, religious delusions, and delusions of guilt, which we suggest can be interpreted as ‘care-harm’. The second factor included delusions of persecution and reference, which we suggest can be interpreted as ‘fairness-injustice’. The third factor included delusions of jealousy, which we suggest can be interpreted as ‘loyalty-betrayal’. The fourth factor included delusions of being controlled, mind reading, thought broadcasting and thought insertion, which we suggest can be interpreted as ‘authority’. Lastly, the fifth factor included somatic delusions, visual, olfactory, and somatic hallucinations, which we suggest may pertain to ‘purity-disgust’. Table 1 provides hypothetical examples outlining the relationship between moral cognition, possible affects, psychotic symptoms, the form and severity or violence, as well as the potential objects of violent acts, thus illustrating the potential of the approach to facilitate more specific violence risk assessments [12, 54].

**Table 1:** *Moral cognitions and sample associated affects, delusions, hallucinations and moral acts*

*Moral cognition*	*Possible affect/ sentiment*	*Potential delusions or hallucinations* *[*51*]*	*Example moral acts*	*Instrumental vs reactive violence*	*Sample object of Violence*
Care – Harm	Compassion Fear Sadness Shame/ Guilt Anger	Grandiose delusions Religious delusions Delusions of guilt *Nihilistic delusions	Mercy killings Altruistic killings Extended suicide Filicide	Primarily instrumental	Children, family members Doctors facilitating abortion
Fairness – Injustice	Anger Fear	Persecutory delusions Delusions of Reference *Mocking voices	Self-defence Revenge violence Spree killings Manslaughter	Primarily reactive	Acquaintances Agencies (e.g. police, schools, etc.)
Ingroup Loyalty – Betrayal	Shame Anger	Delusions of jealousy *Persecution by family *Delusions of misidentification	Crimes of passion Honour killings Patricide Matricide Uxoricide	Primarily reactive	Family members Female partners
Authority – Defiance	Elation/ Happiness Excitement Anger	Delusions of control Mind reading Though insertion Thought broadcasting *Grandiose delusions *Religious delusions *Command hallucinations	Punishments Stranger homicide	Primarily instrumental	Subversives People of perceived lower status
Purity – Degradation	Disgust Anger	Somatic delusions Visual, olfactory, and somatic hallucinations *Critical voices * Religious delusions	Honour killings Punishments Uxoricide Stranger homicide	Primarily instrumental	Paedophiles Hedonists Anti-environmentalists

### Introduction to the current study

In addition to the psychotic symptoms and moral cognitions, the cognitive impairment experienced by many patients with schizophrenia may also play a role in impairing patients’ capacity to make sound judgments regarding moral behaviour when actively psychotic [55–57]. There may also be patients who are so cognitively impaired that they are incapable of moral reasoning [48, 57]. Because we have shown that moral cognition is relevant to acts of serious violence committed by forensic patients [12], and because of the possible association between psychotic symptoms and moral cognition at the time of the offence, we sought to explore moral cognition as a mediating factor for psychotic symptoms and violence. We were primarily interested in mediation modelling, namely whether moral cognition was the missing link or key mediator between psychotic symptoms and acts of violence. Specifically, we sought to examine whether moral cognitions at the time of the offence mediated psychotic symptoms and their ‘relevance’ for violence, the seriousness of violence, and the form of violence.

By ‘relevance’ we mean those symptoms that treating or admitting psychiatrists judged to be causally related to the act of violence although in this study design we used cross-sectional mediation analysis. We reasoned that patients with schizophrenia may only be violent when their loss of contact with reality gives rise to moral cognition. In the context of impaired neurocognitive capacity, this would explain the connection between delusions, hallucinations and violence.

In our earlier exploratory study [12] we concluded that it remains to be shown that the moral dispositions (traits, structures) identified were the cognitions (dynamic state beliefs) relevant to the acts of homicide including delusions, hallucinations and emotions.

We hypothesized:

1. Psychotic symptoms (i.e. delusions and hallucinations), present at the time of the violent act and relevant to acts of violence will not be significantly positively correlated with independently rated forms of violence, such as homicide, seriousness of violence, and instrumental-reactive aggression. By contrast, moral cognitions judged to reflect the patient’s thinking and motivation at the time of the violent act will be significantly positively correlated with forms of violence.
2. Moral cognitions will be significantly associated with the seriousness and form of violence, when controlling for neurocognition and violence proneness.
3. Specific moral cognitions will be significantly positively correlated with specific psychotic symptoms present at the time of the violent act and judged to be ‘relevant’ to the violent act. The correlations will have face validity, by which we mean there will be causal and meaningful connections within the limits of mediation analysis i.e. that the meaningful explanation makes sense.
4. Specific psychotic symptoms will be ‘relevant’ for violence when mediated by specific moral cognitions, and moral cognition may contribute to violence independently of psychotic symptoms.
5. Specific psychotic symptoms will be associated with seriousness and forms of violence when mediated by specific moral cognitions and moral cognitions may contribute to seriousness and forms of violence independently of psychotic symptoms.

## Method

This is a naturalistic cross-sectional study involving a national cohort of forensic patients with schizophrenia or schizoaffective disorder detained at the only forensic hospital, which serves the Republic of Ireland’s population of 4.8 million.

### Setting

The National Forensic Mental Health Service (NFMHS) for Ireland provides specialized care for adults who have a mental disorder and are at risk of harming others. At the time of the study the NFMHS had 94 secure inpatient beds located on a single campus, the Central Mental Hospital (CMH) providing high secure, medium secure, and pre-discharge units.

### Ethics approval and consent to participate

This study was approved by the Research Ethics and Effectiveness Committee of the NFMHS (approval reference number AREE/290814) and complied with the ethical standards of the relevant national and institutional committees on human experimentation and with the Helsinki Declaration of 1975, as revised in 2008. All participants were assessed by their treating consultant psychiatrists as being able to provide informed consent for the study, and all participants gave written, informed consent.

### Participants

**Fig. 1:**
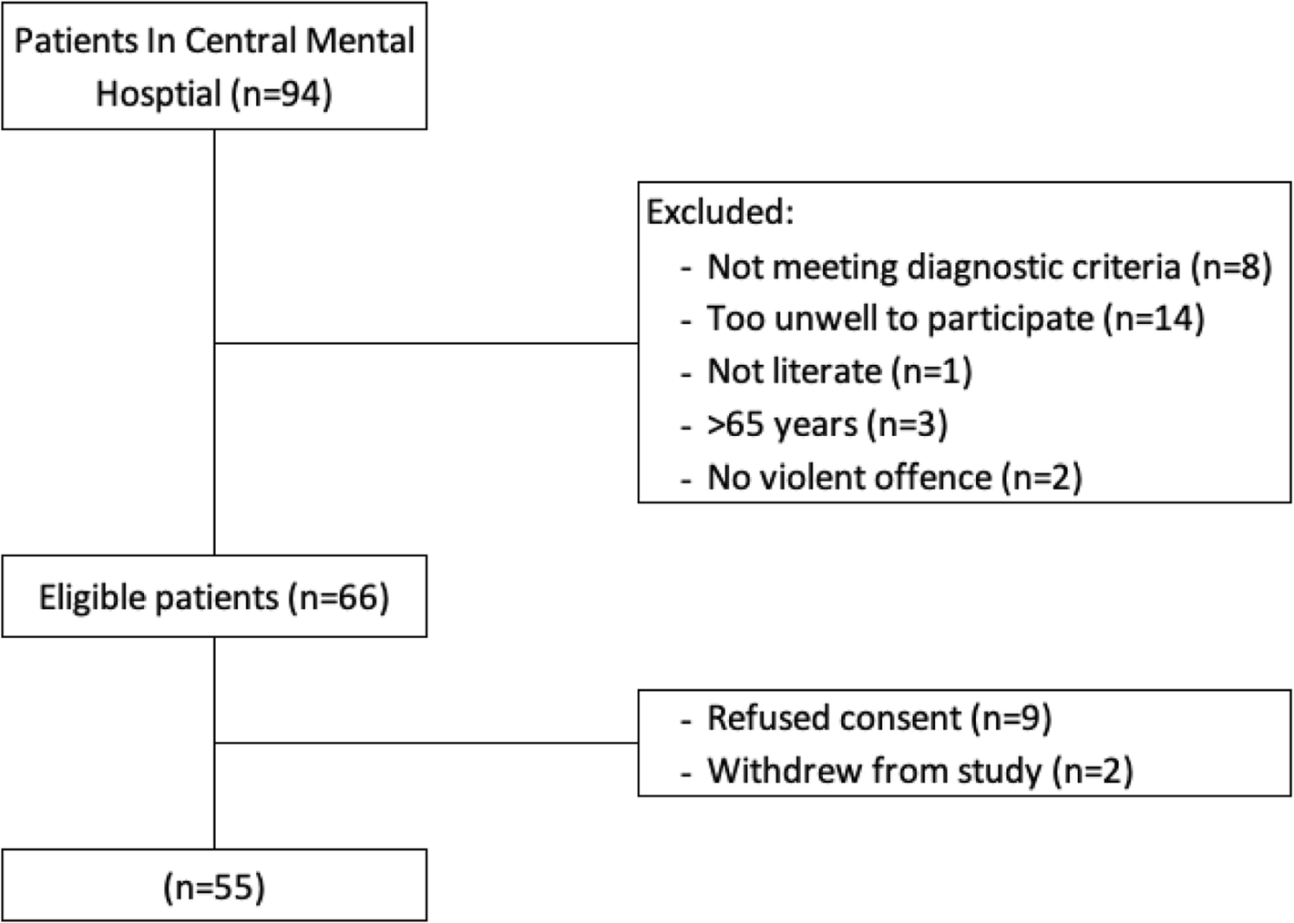
CONSORT Flow diagram

Inclusion criteria were having schizophrenia or schizoaffective disorder assessed using the Structured Clinical Interview for the Diagnostic and Statistical Manual 4th Edition (SCID-I) [58], being judged to be able to provide informed consent by the treating psychiatrist, basic literacy, being less than 65 years of age, and having carried out a violent offence which was proximate to hospitalization to facilitate judgment of a) moral cognition and b) psychotic symptoms. Of the 94 patients detained at the hospital, 55 met the inclusion criteria and participated in the study (Fig. 1; *n =* 55, 58.5% of all inpatients). Of these 55 participants 42 (76.3%) had been found ‘Not Guilty by Reason of Insanity’ (NGRI) by the courts at the time of the study [59]. Of the sample, 27 (49%) had carried out a homicide, and all participants had a history of violence. Of the 55 patients who provided consent, 51 patients completed the self-report measure of moral cognition relevant to the offence. Four patients denied or claimed no memory of the offence or denied having committed the offence when presented with the moral cognition index offence questionnaire, or were discharged prior to completing the moral cognition index offence questionnaire.

### Measures

#### Validity

No single measure of validity or malingering could have covered all the possible sources of bias. In addition to multiple one to one interviews with the patients and other informants, independent expert witnesses (consultant psychiatrists) and treating consultant psychiatrists in all cases read the ‘book of evidence’, the sworn statements of witnesses at the trial, and were able to read the family practice medical notes, correspondence and investigation results and psychiatric notes. Correspondence and investigation results covered all of adult life in most cases. All of the participants were in-patients in the forensic hospital for up to a year prior to trial, and subject to continuous nursing observation and psychological and multidisciplinary assessments. This combination of sources and assessments was taken as an appropriate way of checking against malingered mental illnesses. The use of witness statements, multiple informants and multiple raters helped to minimise the possibility that any bias was introduced in the measurement of moral cognition.

#### Violence

Homicide was defined as any non-accidental death caused by a patient in accordance with the definition provided by “those interpersonal assaults and other acts directed against another person (for example, poisonings) that occur outside the context of warfare and prove fatal” [34]. Homicide was coded using all available information from patient files, including in all cases charge sheets, book of witness statements presented at trial, and psychiatric court reports.

Other violence was defined as a court conviction or finding of NGRI for violence towards a person other than homicide, or as a documented act of interpersonal violence causing harm or threatened harm to a person. Verdicts at trial were known for all patients.

All the violent acts were also coded independently by a clinical psychologist using Cornell’s (1996) instrumental-reactive aggression coding scheme [46], again using all the available information. Cornell’s scheme allows for a form of violence, namely instrumental-reactive violence, to be coded on a four-point scale. Scores of ‘4’ represent clearly instrumental aggression involving goal setting and planning, scores of ‘3’ represent primarily instrumental aggression with some expressive qualities, scores of ‘2’ represent primarily reactive hostile aggression with some instrumental qualities and scores of ‘1’ represent clearly reactive hostile aggression.

The scheme also facilitates the coding of the seriousness of violence as distinct from homicide on a 7-point scale with scores of ‘7’ representing extreme homicide involving multiple killings and mutilation, ‘6’ homicide, ‘5’ severe injury, ‘4’ serious injury, ‘3’ minor injury, ‘2’ assault without injury, and scores of ‘1’ representing no assault e.g. threatened with a weapon. Need for therapeutic security at the time of admission was assessed using the Dangerousness, Understanding, Recovery, Urgency, Manual triage security scale (DUNDRUM-1) [60, 61].

### Assessment of psychotic symptoms at the time of the violent act

Independent of the violence ratings, clinicians who were involved in admitting or treating the patient at the point of admission were asked to complete a retrospective Schedule for the Assessment of Psychotic Symptoms (SAPS) [62] concerning the mental state at the time of the offence, using all available information including mental state assessment nearest to the time of the offence from patient files and court reports. The SAPS measures twelve forms of delusions each on a 6-point Likert scale (0–5) with ‘0’ representing none, ‘1’ questionable, ‘2’ mild, ‘3’ moderate, ‘4’ marked and ‘5’ severe. SAPS scores of > 2 indicated significant symptoms with functional impairment. Forms of delusions measured by the SAPS include delusions of persecution, jealousy, guilt or sin, grandiosity, religion, somatic, reference, passivity/control, broadcasting, thought insertion, and thought withdrawal. In addition to this, we added nihilistic delusions and delusions of misidentification or ‘doubles’ e.g. Capgras/ Fregoli.

The SAPS also measures six forms of hallucinations: auditory hallucinations (such as noises or sounds) voices commenting, voices conversing, somatic or tactile hallucinations, olfactory hallucinations and visual hallucinations. We added the category of ‘voices criticizing’ and ‘command hallucinations’ because of their potential relevance for violence [63].

In addition to assessing the presence of symptoms, we also asked the treating and admitting psychiatrists (*n* = 3) to assess the ‘relevance’ of the symptoms using the SAPS for the violent act using the same six-point Likert Scale, again using all relevant information. We asked whether in their judgement specific symptoms were causally related to the violent act. SAPS total score had Cronbach’s alpha = 0.65.

### Assessment of moral cognition at the time of a violent offence

Independent of the violence ratings, and independent of the treating or admitting psychiatrist, an ‘expert witness’ (*n* = 10) namely a consultant psychiatrist who gave expert evidence at the trial of each patient concerning the violent offence which led to hospitalization, made a retrospective assessment of moral cognition present at the time of the violent act, using all the available information. Moral cognitions are those thoughts congruent with one of the five foundations derived from Haidt’s moral foundations theory [39]. These expert witnesses were instructed to consider the patient’s mindset and motivation at the time of the offence and then to rate how well the five domains of moral cognitions, derived from Haidt’s moral foundation theory, reflected the patient’s thinking at the time of the violent offence (See Appendix A) using (1–6) Likert scales. Moral cognitions involving ‘care-harm’, ‘fairness-injustice’, ‘loyalty-betrayal’, ‘authority’ and ‘purity-disgust’ were all rated in this way. Ratings of ‘6’ and ‘1’ were tethered by ‘completely agree’ and ‘completely disagree’ to facilitate dichotomization. Scores of ‘4’, ‘5’, and ‘6’ indicated that the expert witness clearly considered their index offence or violent act that led to hospitalization to be clearly motivated by moral cognitions; scores of ‘2’, and ‘3’ indicated that an expert was less clear whether the moral cognition played a role. Very low scores i.e. ‘completely disagree’, indicated absence of that moral cognition, not the presence of its opposite. To establish the reliability of this approach, we also asked the patients themselves to rate their violent offence in accordance with the five domains of moral cognitions derived from the five moral foundations outlined by Haidt’s moral foundation theory on a 6-point Likert scale.

### Neurocognition

We assessed the cognitive abilities of patients with the composite score, MATRICS Consensus Cognitive Battery (MCCB) [64] for schizophrenia. The MCCB assesses seven areas of cognition affected by schizophrenia: processing speed; attention/ vigilance; working memory; verbal learning; visual learning; reasoning and problem solving; and social cognition. A composite score is produced by aggregation.

### Assessment of violence proneness

The Historical Clinical Risk Management-20 version 2 (HCR-20) [65] measures risk of violence. This was rated by forensic psychiatry higher trainees, blind to other assessments. The historical scale of the HCR-20 (HCR-20-H) contains ten ‘static’ items. The HCR-20 also includes 5 current clinical items and 5 future risk items. All items are weighted equally. Only the HCR-20-H items were used as a means of controlling for violence proneness as these are the most stable and best predictors.

### Statistical analysis

Data were analysed using SPSS version 24 (IBM, 2013). All statistical tests were two-tailed, alpha was set at α < .05. Inter-rater reliability was calculated for psychotic symptoms, moral cognitions, and the seriousness and form of violence using intraclass correlations (ICC) using the method of absolute agreement, and one-way random effects models. Inter-rater reliability for psychotic symptoms using the SAPS was calculated using admitting or treating clinician (the criterion), and expert witnesses. Inter-rater reliability for moral cognitions was calculated using expert witnesses (the criterion), treating or admitting psychiatrists, and also an independent clinician in addition to patients’ own self-rating. Inter-rater reliability was calculated for the seriousness and form of violence using Cornell’s instrumental-reactive aggression coding scheme by randomly selecting 20% of cases. ICC values less than 0.5 are indicative of poor reliability, 0.5 to 0.75 moderate reliability, 0.75 to 0.9 good reliability and greater than 0.9 excellent reliability [66]. Associations between presence and relevance of delusions and moral cognitions were assessed using binomial probability for non-random association.

Omnibus MANOVA tests were used to examine the robustness of associations. Wilk’s lambda statistic was chosen because the hypothesis degrees of freedom were greater than 1 in each case and because other conditions were met for preferring it. Eta^2^ statistic expresses the proportion of variance explained by the model.

In keeping with other preliminary and novel studies exploring the relationship between psychotic symptoms and violence we chose to forgo Bonferroni corrections due to the risk of type II errors [11]. This study uses omnibus tests and boot strapped confidence intervals to mitigate the risk of type I error. Because all of the participants had been violent and had experienced psychotic symptoms at the time of their violent act, we only examined positive correlations between psychotic symptoms, moral cognitions, and the seriousness and form of violence, with the exception of reactive aggression, which is negatively associated with instrumental aggression.

We reported frequencies of psychotic phenomena, both present and judged relevant to the violent act (rated by treating and admitting psychiatrists) and frequencies of moral cognitions (rated by expert witnesses and the patients themselves using dichotomized ratings), in addition to the means and standard deviations for expert and patient rated moral cognitions.

Spearman correlations were used to analyse associations between psychotic symptoms (i.e. delusions and hallucinations), moral cognition, and forms of violence such as homicide, seriousness of violence and instrumental-reactive aggression. Positive correlations between moral cognitions, delusions and homicide were used to select variables in regression models.

Binary logistic regression was used to examine the relationship between moral cognition and homicide the only binary outcome variable, whilst controlling for neurocognitive impairment using the MCCB composite score and violence proneness using the HCR-20 H-scale. Linear regression was used to examine the relationship between moral cognition, severity of violence a continuous outcome variable and instrumental-reactive aggression also a continuous outcome variable, whilst also controlling for neurocognitive impairment and violence proneness. All results are presented as both Odds ratios and Betas i.e. unstandardized effect sizes with 95% confidence intervals.

Mediation analysis [67] was performed using Hayes’ SPSS process macro model 4 with 10,000 bootstrapped samples to calculate 95% confidence intervals surrounding the parameter. Mediation is a statistical technique for exploring potential causal relations by clarifying pathways. A mediating variable (M) explains how an independent variable (X) affects a dependent or outcome variable (Y). The Hayes’ process marcro uses linear regression for continuous variables and binary logistical regression for binary outcomes [67]. Variables can be entered into mediation models to satisfy the criteria for causal inference: temporal ordering, association, and controlling for confounding variables. Use of boot-strapped confidence intervals protects to some extent against type I error.

Mediation modelling can be used to clarify the direct effect of X on Y before mediation, as well as the effect of X on Y after mediation, the effect of M the mediating variable on Y controlling for X, in addition to the indirect effect of X on Y via M. The primary effect of interest in mediation models is the indirect effect according to Preacher and Kelly [68]. Since the indirect effect cannot be converted to a classical effect size measure such as Cohen’s d new measures of indirect effect have been developed [68]. However according to Preacher and Kelley [68] effect sizes suggested for mediation analysis should be based on three criteria a) use of a meaningful metric b) be amenable to the construction of confidence intervals c) and should be independent of sample size. Within our mediation analyses all variables are based on a meaningful metric such as the ‘the relevance of symptoms for acts of violence’ using the SAPS, the occurrence of homicide (a binary outcome), the severity of violence as measured using the Cornell instrumental-reactive scale (where one unit for example distinguishes between homicide and multiple homicide), as well as whether the violence was instrumental or reactive, again measured using the Cornell scale. In keeping with the American Psychological Task force for Statistical inference, because our units of measurement are meaningful at a practical level we opted for an unstandardized measure of effect size over a standardized measure as recommended by Wilkinson [69]. The indirect effect expressed by B can therefore be read as: one unit change in the independent variable (X) will lead to the corresponding indirect effect of X on Y via M. In every case we provide the total amount of variance accounted for by the model.

### Model construction

MANOVA was used as a robust omnibus test with the five moral cognitions as the dependent variables (each entered separately), the fixed factor was either the severity or the form of violence, or total psychopathology measured as SAPS total score.

The relationship between psychotic symptoms judged to be present at the time of the violent act and the relevance of the same psychotic symptoms to the violent act was investigated using mediation via the independent rating of moral cognition.

We reasoned that the relevance of psychotic phenomena for acts of violence is determined by the extent to which they have a moral component. It follows that in this model, moral cognitions do not cause delusions and hallucinations but may arise from them. Therefore, to satisfy temporal ordering, delusions or hallucinations were entered as the independent variable (X), moral cognition was entered as the mediator (M), and delusions judged to be relevant for the outcome as the dependent variable (Y). Only moral cognitions positively associated with the independent variables will be entered into the model to satisfy the criterion of association and to avoid spurious associations. For mediation to be demonstrated, it must be shown that the independent variables associated with the dependent variable are altered by including the mediator, for example eliminating, generating, strengthening, or reversing an association [8].

In addition to these mediation analyses, we explored if specific moral cognitions could independently contribute to the specific delusions and hallucinations judged ‘relevant’ when controlling for their presence using regression. Similarly, we explored whether specific moral cognitions and specific psychotic symptoms were related to homicide or other violent outcomes.

To examine whether expert-rated moral cognitions mediated the relationship between psychotic symptoms and forms of violence, such as homicide (a binary outcome variable), severity of violence and reactive instrumental aggression, we also used mediation analysis with 10,000 bootstrapped samples.

## Results

### Sample characteristics

Of the sample (*n* = 55), 49 (89%) were male. The mean age was 40 (*SD* = 9.7) years, the mean age at the time of the violent offence was 32 years, the mean item score on the DUNDRUM-1 nine item scale was 2.89 (*SD* 0.46, range 1.8 to 3.8); the mean total score of the HCR-20 was 25 (*SD =* 5.66), and for the historical scale (HCR-20-H), 13 (*SD =* 3.11). The mean total score on the SAPS was 17.89 (*SD* 11.14, 0 to 53) as rated by treating or admitting psychiatrists. At the time of the study, the mean length of stay within the forensic hospital was 5 years (*SD* = 4.96). There was no significant difference between the homicide group and other violence groups on the HCR-20-H, or for the total HCR-20 score (*t* = 1.5, *df* = 53, *p* = .140; *t* = 1.098, *df* = 53, *p* = .277). There was no significant difference between the homicide group and other violence groups on SAPS total (*t* = 1.9, *df* = 53, *p* = .854) The mean MCCB [53] score was 26 (*SD* 12.13, range 0 to 54; (t-scores have a mean of 50, *SD* = 10). There was no significant difference between the homicide and other violence groups on the MCCB (*t* = 1.496, *df* = 53, *p* = .141). There was no significant association between gender and homicide (Chi-squared .832, *df* = 1, *p* = 0.362). There was no significant association between NGRI and homicide (Chi-squared .292, *df* = 1, *p* = .589).

Of the 55 patients who participated in the study, 10 carried out a violent act which was judged to be clearly instrumental, 7 carried out a violent act which was primarily instrumental but with some reactive qualities, 14 with primarily reactive hostile aggression but some instrumental qualities, and 24 with clearly reactive hostile aggression. Overall, 31% of patients carried out an act of violence which was primarily instrumental and 69% carried out an act of violence which was primarily reactive. The mean score for instrumental violence using the Cornell’s scale was 2.04 (*SD* = 1.14*).*

Of the 55 patients who participated, six obtained a score of ‘7’ representing extreme homicide involving multiple killings and mutilation. 22 obtained a score of ‘6’ representing a homicide, three obtained a score of ‘5’ representing severe injury, eight obtained a score of ‘4’ representing serious injury, six obtained a score of ‘3’ representing minor injury, three obtained a score of ‘2’ representing assault without injury, and seven obtained a score of ‘1’ representing threatened with a weapon. The mean score for the severity of violence using the Cornell scale was 4.58 (*SD* = 1.95).

### Inter-rater reliability

Table 2 shows that intra-class correlations between expert witnesses, treating or admitting clinicians, and an independent research clinician for ratings of moral cognitions at the time of the violent act were all moderate to good.

**Table 2:** *Inter-rater reliability using intra-class correlations (ICC) all as compared with the expert witness at time of trial*

	Expert witness (*n* = 55)		Admitting or treating clinician (*n* = 50)			Patient self-rating (*n* = 51)			Independent research clinician (*n* = 55)			Average ICC
	*n*	*%*	*ICC*	*n*	*%*	*ICC*	*n*	*%*	*ICC*	*n*	*%*	
Care-harm	11	20.0	.59	9	17.6	.58	20	39.2	.77	11	20.0	.80
Fairness-injustice	35	63.6	.95	34	66.7	.51	24	47.1	.64	36	65.0	.79
Loyalty-betrayal	30	54.5	.75	23	45.1	.42	23	45.1	.71	29	52.0	.75
Authority	17	30.9	.73	13	25.5	.75	18	35.3	.83	17	30.9	.89
Purity-disgust	14	25.5	.69	15	29.4	.52	10	19.6	.40	4	7.3	.64
Average			.74			.56			.67			.77

Table 3 shows the intra-class correlations between treating or admitting clinicians and expert witnesses for ratings of symptoms at the time of the violent act were variable, with ICC greater than 0.5 (moderate) for all except delusions of reference, experiences of being controlled, mind reading, thoughts broadcasted, thought insertion, olfactory hallucinations, somatic and tactile hallucinations, hearing noises or sounds, voices commenting and voices criticizing. Table 3 also shows the incidence of symptoms, as judged independently by treating or admitting clinicians and consultant psychiatrists acting as expert witnesses.

**Table 3:** *SAPS ratings of patient psychopathology at the time of the violent act*

	^a^ICC (treating clinicians v expert witnesses)	^b^Total (treating clinician’s ratings)		^b^Relevant (treating clinicians)	
		*n*	*%*	*n*	*%*
Grandiose	.84	11	20	11	20.0
Religious	.89	17	30.9	17	30.9
Guilt	.55	3	5.4	3	5.4
Persecution	.63	41	74.5	39	71
Reference	.26	13	23.6	9	16.3
Jealousy	.76	4	7.2	3	5.4
Misidentification	.72	10	18.1	8	14.5
Being controlled	.19	10	18.1	7	12.7
Mind reading	.46	4	7.2	2	3.6
Thought broadcast	.17	2	3.6	1	1.8
Thought insertion	.42	1	1.8	0	.0
Thought withdrawal	.58	1	1.8	1	1.8
Somatic	.64	5	9.0	4	7.2
Visual hallucinations	.57	7	12.7	5	9.0
Olfactory hallucinations	.40	3	5.4	1	1.8
Somatic hallucinations	.41	6	10.9	2	3.6
Nihilistic	.88	1	1.8	1	1.8
Noises and sounds	.03	8	14.5	5	9.0
Voices commenting	.06	8	14.5	5	9.0
Command hallucinations	.68	15	27.2	15	27.2
Voices conversing	.35	10	18.1	5	7.2
Voices criticising	.46	6	10.9	4	7.2

^a^*Inter-rater reliability (intra-class correlations, ICC) are calculated by comparing the expert witness and the treating or admitting clinician for total symptoms present*

^b^*Total numbers positive for each symptom and symptoms rated as ‘relevant’ to the violent act were scored by treating or admitting clinicians*

### Inter-rater reliability for the Cornell instrumental-reactive aggression coding scheme (*n* = 11)

The ICC for instrumental reactive was .78 (good), and the ICC for severity of violence was .97 (excellent).

### Main outcomes

Table 3 shows the prevalence of symptoms (SAPS) and relevance of symptoms at the time of the violent act as rated by treating or admitting clinicians.

The expert witnesses judged that none of the moral cognitions were clearly present (moral cognitions scoring > 3) in one case (1.8%), one moral cognition was present in 16 cases (29.1%), two moral cognitions were present in 24 cases (43.6%), three moral cognitions were present in 13 (23.6%) and four moral cognitions were present in one case (1.8%). No experts found all five domains of moral cognition to be clearly present concurrently in any one case.

Table 2 shows the numbers clearly present for each of the domains of moral cognitions at the time of the violent act, as rated by expert witnesses, patients’ self-ratings, and an independent research clinician.

For expert witnesses, the mean scores and standard deviations for the moral foundations were as follows: ‘care-harm’, 2.03 (*SD* = 1.74), ‘fairness-injustice’, 3.98 (*SD* = 2.01), ‘loyalty-betrayal’, 3.47 (*SD* = 2.07), ‘authority’, 2.65 (*SD* = 2.05), and ‘purity-disgust’, 2.27, (*SD* = 1.76).

Of the 51 patients that completed the self-report measure, six indicated that no moral foundation was clearly present (11.7%), 17 indicated that one domain of moral cognition was present (33.3%), 15 indicated that two domains of moral cognition were present (29.4%), five indicated that three domains of moral cognitions were present (9.8%), seven indicated that four domains of moral cognitions were present (13.7%), and one indicated that all five domains of moral foundations were clearly present (2%).

For patients, the mean scores and standard deviations for the five moral cognitions were as follows: ‘care-harm’, 2.92 (*SD* = 2.09), ‘fairness-injustice’, 3.17 (*SD* = 2.08), ‘loyalty-betrayal’, 3.11 (*SD* = 2.15), ‘authority’, 2.98 (*SD* = 2.27), and ‘purity-disgust’, 2.01, (*SD* = 1.71).

**Hypothesis 1:**

**H1.1 Psychotic symptoms i.e. delusions and hallucinations, present at the time of the violent act and relevant to acts of violence will** **not** **be significantly positively correlated with independently rated forms of violence, such as homicide, seriousness of violence, and instrumental-reactive aggression.**

**H1.2 By contrast, moral cognitions judged to reflect the patient’s thinking and motivation at the time of the violent act will be significantly positively correlated with forms of violence.**

Table 4 shows three MANOVA omnibus tests for relationships between all symptoms, all moral cognitions, and severity and forms of violence. In model 1, total SAPS psychopathology score is significantly related to moral cognitions (*p* = 0.041, Eta^2^ = 0.632). In model 2, instrumental-reactive violence is related to the five moral cognitions (*p* < 0.001, Eta^2^ = 0.27). In model 3, severity of violence is significantly related to five moral cognitions (*p* = 0.032, Eta^2^ = 0.17).

**Table 4:** MANOVA omnibus tests for relationships between total symptom severity scores (SAPS), five moral cognition scores and qualities of violence (instrumental-reactive, severity)

Model	Fixed factor	Dependent variables	Wilk’s lambda	F	df	p	Eta^2^
1	SAPS-total	Five moral cognitions	0.008	1.371	140,113	0.041	0.623
2	Instrumental-reactive	Five moral cognitions	0.384	3.599	15,147	0.000	0.273
3	Severity of violence	Five moral cognitions	0.384	1.603	30,178	0.032	0.174

H1.1. The only finding that was contrary to the hypothesis that psychotic symptoms were not correlated with the seriousness of violence and forms of violence, was that delusions of guilt were positively correlated with instrumental aggression (*r* = .304, *n* = 55, *p* = .024). Homicide was not positively correlated with SAPS-rated delusions or hallucinations although delusions of misidentification or doubles approached significance (*r* = .255, *n* = 55, *p* = .060). Seriousness of violence was not positively correlated with psychotic symptoms present or judged to be ‘relevant’ to the act of violence. Instrumental or reactive violence was not positively correlated with any other psychotic symptoms.

H1.2: We found that certain moral cognitions were correlated with seriousness of violence and with forms of violence. Expert rated ‘loyalty-betrayal’ was positively correlated with homicide (*r* = .310, *n* = 55, *p* = .021). Expert rated ‘loyalty-betrayal’ was also positively correlated with seriousness of violence (*r* = .289, *n* = 55, *p* = .032). Expert rated ‘fairness-injustice’ was negatively correlated with instrumental aggression (*r* = −.424, *n* = 55, *p* = .001) i.e. positively with reactive violence. Expert rated ‘loyalty-betrayal’ was negatively correlated with instrumental aggression (*r* = −.510, *n* = 55, *p* = .000) as was expert-rated ‘purity-disgust’ (*r* = −.329, *n* = 55, *p* = .014) i.e. both were positively correlated with reactive aggression.

**Hypothesis 2: Moral cognitions will account for forms of violence, when controlling for neurocognition and violence proneness (HCR-20).**

Table 5 shows that for each unit change in the rating of ‘loyalty-betrayal’ (rated 1–6) as relevant to the offence, homicide was increased in likelihood by OR of 1.392 (95% CI 1.055–1.837) and this remained significant when adjusted for neurocognition (MCCB) scores or violence proneness (HCR-20-H) scores. No other domain of moral cognition was significantly related to the likelihood of homicide.

**Table 5:** *Homicide* versus *other violence: binary logistical regression*

Moral cognition	^a^OR	CI 95%		^a^OR adjusted MCCB	CI 95%		^a^OR adjusted HCR-20-H	CI 95%	
		Lower Upper			Lower Upper			Lower Upper	
Care	.794	.571	1.104	.771	.546	1.088	.769	.547	1.081
Fairness	.973	.746	1.268	.966	.737	1.267	.979	.747	1.283
Loyalty	*1.392*	*1.055*	*1.837*	*1.451*	*1.079*	*1.950*	*1.411*	*1.059*	*1.879*
Authority	1.042	.803	1.350	1.025	.785	1.338	1.028	.789	1.340
Purity	.858	.629	1.169	.823	.596	1.138	.854	.623	1.171
Cornell’s seriousness of violence scale: linear regression									
Moral cognition	Beta	CI 95%		Beta adjusted MCCB	CI 95%		Beta adjusted HCR-20-H	CI 95%	
		Lower Upper			Lower Upper			Lower Upper	
Care	−.257	−.559	.044	−.267	−.570	.036	−.277	−.575	.021
Fairness	−.052	−.320	.216	−.055	−.324	.214	−.047	−.312	.219
Loyalty	*.260*	*.017*	*.517*	*.275*	*.024*	*.526*	*.263*	*.016*	*.511*
Authority	−.026	−.288	.236	−.036	−.300	.229	.-.039	.-.299	.222
Purity	.002	−.304	.307	−.013	−.321	.296	.003	.-.300	.305
Cornell’s instrumental-reactive aggression: linear regression									
Moral cognition	Beta	CI 95%		Beta adjusted MCCB	CI 95%		Beta adjusted HCR-20	CI 95%	
		Lower Upper			Lower Upper			Lower Upper	
Care	.054	−.126	.235	.058	−.124	.240	.060	.-.122	.242
Fairness	*−.283*	*−.419*	*−.147*	*−.282*	*−.419*	*−.145*	*−.285*	*−.421*	*−.149*
Loyalty	*−.269*	*−.402*	*−.136*	*−.274*	*−.407*	*−.140*	*−.268*	*−.402*	*−.135*
Authority	*.162*	*.015*	*.309*	*.168*	*.020*	*.316*	*.167*	*.019*	*.314*
Purity	−.165	−.337	.008	−.160	−.335	.015	−.165	−.338	.008

^a^*OR* odds ratio

^b^Bold: confidence interval does not move from positive to negative

For each unit change in the rating of ‘loyalty-betrayal’ (1–6) as relevant to the offence, the score on the Cornell seriousness of violence scale increased by one unit i.e. more serious. This also remained significant even when adjusted for neurocognition (MCCB) or violence proneness (HCR-20-H).

Table 5 also shows that for each unit change in the rating of ‘fairness-injustice’ or ‘loyalty-betrayal’ (1–6) as relevant to the offence, the score on the Cornell instrumental-reactive aggression scale decreased i.e. more reactive, and that for each unit change in the rating for authority (1–6) as relevant to the offence, the Cornell reactive-instrumental violence scale increased i.e. more instrumental. This also remained significant even when adjusted for neurocognition (MCCB) or violence proneness (HCR-20-H).

**Hypothesis 3: Specific moral cognitions will be significantly positively correlated with specific psychotic symptoms present at the time of the violent act or judged to be relevant to the violent act. The correlations will have face validity, by which we mean there will be causal and meaningful connections** **within the limits of mediation analysis** **i.e. that the meaningful explanation makes sense.**

We found that specific moral cognitions rated by ‘expert witnesses’ correlated positively with specific delusions or hallucinations. The moral cognition involving ‘care-harm’ correlated positively with religious delusions (*r* = .421, *n* = 55, *p* = .001); ‘relevant’ religious delusions (*r* = .443, *n* = 55, *p* = .001); grandiose delusions (*r* = .281, *n* = 55, *p* = .038); and ‘relevant’ grandiose delusions (*r* = .279, *n* = 55, *p* = .039). ‘Care-harm’ also correlated with ‘relevant’ delusions of guilt (*r* = .438, *n* = 55, *p* = .001), but not with other delusions or hallucinations. All are in keeping with the factor analysis of Peralta [51] (Table 1).

The moral cognitions involving ‘fairness-injustice’ correlated positively with delusions of persecution (*r* = .391, *n* = 55, *p* = .003); ‘relevant’ persecutory delusions (*r* = .427, *n* = 55, *p* = .001), somatic delusions (*r* = .316, *n* = 55, *p* = .019); ‘relevant’ somatic delusions (*r* = .304, *n* = 55, *p* = .024); delusions of mind reading (r = .282, n = 55, *p* = .037); ‘relevant’ delusions of mind reading (*r* = .282, *n* = 55, *p* = .037); somatic or tactile hallucinations (*r* = .270, *n* = 55, *p* = .046); ‘relevant’ somatic or tactile hallucinations (*r* = .304, *n* = 55, *p* = .024), and no other delusions or hallucinations.

The moral cognitions involving ‘loyalty-betrayal’ correlated positively with delusions of persecution (*r* = .355, *n* = 55, *p* = .008); and ‘relevant’ delusions of persecution (*r* = .417, *n* = 55, *p* = .002) and no other delusions or hallucinations.

The moral cognitions involving ‘authority’ correlated positively with religious delusions (*r* = .480, *n* = 55, *p* = .000); relevant religious delusions (*r* = .509, *n* = 55, *p* = .000); command hallucinations (*r* = .310, *n* = 55, *p* = .021); and relevant command hallucinations (*r* = .323, *n* = 55, *p* = .016).

The moral cognition involving ‘purity-disgust’ was significantly positively correlated with relevant voices criticizing (r = .328, n = 55, *p* = .015) but no other delusions or hallucinations.

There was a tendency for correlations between symptoms and moral cognitions to be stronger where the symptom was judged ‘relevant’ to the violent act. Out of 11 cases where there was a significant correlation; in 8 cases the ‘relevant’ symptom had stronger correlations with moral cognitions, 1 case was tied, and in 2 cases symptoms that were present at the time of the act had stronger correlations than those that were judged by treating psychiatrists to be ‘relevant’ to the act. Using the binomial probability theorem [70, 71] and assuming an equal probability for present or ‘relevant’ symptoms to have stronger correlations with moral cognitions the Binomial probability was *p* = 0.043. This finding indicates that symptoms judged to be ‘relevant’ by the treating or admitting psychiatrists were more likely to have a stronger correlation with specific moral cognitions than those symptoms which were judged only to be present.

**Hypothesis 4: Specific psychotic symptoms will be** **relevant** **for violence when mediated by specific moral cognitions and moral cognition may contribute to violence independently of psychotic symptoms.**

Table 6 shows the results of mediation analysis with models derived as described above. We found evidence that specific moral cognitions mediated the relationship between the presence of specific psychotic symptoms and their ‘relevance’ for acts of violence.

**Table 6:** The mediating effect of moral cognition on delusions / hallucinations (X) and relevance for the violent act)

*n = 55*			C1 direct effect of X (SAPS) on Y before mediation			C2 direct effect of X (SAPS) on Y after mediation			A: indirect effect of X (SAPS) on Y mediated via M (Expert rated morality)			B: direct effect of M (Expert Rated Morality) on Y adjusted for X (SAPS)		
	*R*^*2*^	*p*	*Unstandardized effect size or OR*	*95% CI*		*Unstandardized effect size*	*95% CI*		*Unstandardized effect size*	*95% CI*		*Unstandardized effect size*	*95% CI*	
M is care-harm, X are delusions Y is whether the delusions or hallucinations were relevant to the violent act														
X = Delusions of guilt Y = Relevance	.999	.000	**.867**	**.767**	**.967**	9.900	-1163.928	1183.730	***3.128***	***1.854***	***6.690***	***3.699***	***-575.590***	***582.988***
X = Religious delusions Y = Relevance	.960	.000	.***962***	**.908**	**1.016**	***.952***	***.891***	***1.012***	.010	-.0097	.060	.030	-.048	.108
X = Grandiose Y = Relevance	.949	.000	**.839**	**.762**	**.915**	**.835**	**.755**	**.914**	.004	-.0212	.048	.015	-.065	.097
M is fairness-injustice, Y is whether the delusions or hallucinations were relevant to the violent act														
X = Persecutory delusions Y = Relevance	.930	.000	***.953***	***.846***	***1.062***	***.917***	***.807***	***1.027***	*.035*	*-.001*	.139	***.125***	***.010***	***.239***
X = Somatic delusions Y = Relevance	.934	.000	***.870***	***.779***	***.961***	***.874***	***.777***	***.971***	-.004	-.022	.003	-.008	-.067	.051
X = Delusions of mind reading Y = Relevance	.897	.000	***.634***	***.548***	***.720***	***.635***	***.546***	***.724***	-.001	-.023	.010	-.002	-.056	.050
X = Somatic/tactile hallucinations Y = Relevance	.745	.000	***.404***	***.302***	***.505***	***.392***	***.288***	***.469***	***.011***	***.000***	***.039***	.034	-.031	.110
M is loyalty-betrayal, Y is whether the delusions or hallucinations were relevant to the violent act														
X = Persecutory delusions Y = Relevance	.867	.000	***.953***	***.844***	***1.062***	***.915***	***.805***	***1.024***	***.038***	***.005***	***.135***	***.130***	***.019***	***.241***
X = Delusions of doubles Y = Relevance	.919	.000	***.911***	***.804***	***1.019***	***.916***	***.806***	***1.026***	-.004	-.038	.005	-.018	-.105	.067
M is authority, Y is whether delusions or hallucinations were relevant to the violent act														
X = Religious delusions Y = Relevance	.980	.000	***.962***	***.908***	***1.016***	***.960***	***.893***	***1.027***	.002	-.0338	.055	.004	-.068	.077
X = Command hallucinations Y = Relevance	.961	.000	***.868***	***.798***	***.938***	***.892***	***.814***	***.970***	-.023	-.0813	.007	-.053	-.132	.026
M is sanctity -degradation, Y is whether delusions or hallucinations were relevant to the violent act.														
X = Auditory hallucinations Y = Relevance	.898	.000	***.863***	***.783***	***.943***	***.857***	***.773***	***.940***	.006	-.0088	.035	.020	-.048	.089

Bold: confidence interval does not move from positive to negative

Religious delusions and grandiose delusions correlated with moral cognitions involving ‘care-harm’; however, there was no mediating relationship between ‘care-harm’ and the ‘relevance’ of these delusions to violence. Although delusions of guilt were not correlated with ‘care-harm’, it significantly contributed to the ‘relevance’ of delusions of guilt to acts of violence when controlling for their presence.

There was no mediating relationship between ‘fairness-injustice’, persecutory delusions and the ‘relevance’ of persecutory delusions to violence. However, ‘fairness-injustice’ made an independent contribution to the ‘relevance’ of persecutory delusions to acts of violence, when controlling for persecutory delusions.

Although somatic delusions and delusions of mind reading were correlated with ‘fairness-injustice’, there was no mediating relationship between ‘fairness-injustice’ and the relevance of these delusions to acts of violence.

The relationship between somatic or tactile hallucinations and their relevance for violence was completely mediated by moral cognitions regarding ‘fairness-injustice’.

The relationship between persecutory delusions and their ‘relevance’ to violence was completely mediated by ‘loyalty-betrayal’, with ‘loyalty-betrayal’ also making an independent contribution to the model. Finally, although religious delusions and command hallucinations were correlated with ‘authority’, their ‘relevance’ to acts of violence was not mediated by ‘authority’.

**Hypothesis 5: Specific psychotic symptoms will be associated with seriousness and forms of violence when mediated by specific moral cognitions and moral cognitions may contribute to seriousness and forms of violence independently of psychotic symptoms.**

We found evidence that specific moral cognitions mediated the relationship between psychotic symptoms and the seriousness and form of violence (Table 7).

**Table 7:** The mediating effect of moral cognition on homicide, seriousness of violence and reactive-instrumental violence

*n = 55*	Homicide		C1 direct effect of X (SAPS) on Y before mediation			C2 direct effect of X (SAPS) on Y after mediation			A: indirect effect on X (SAPS) on Y mediated via M (Expert rated morality)			B: direct effect of M (Expert Rated Morality) on Y adjusted for X (SAPS)		
	*R*^*2*^	*p*	*Unstandardized effect size*	*95% CI*		*Unstandardized effect size*	*95% CI*		*Unstandardized effect size*	*95% CI*		*Unstandardized effect size*	*95% CI*	
M is loyalty-betrayal, Y is homicide														
X = Persecutory delusions	.320	.000	-.260	-.534	.013	-.498	-.860	-.136	***.163***	***.015***	***.456***	***.555***	***.185***	***.926***
X = Delusions involving doubles	.215	.007	**.438**	**.0188**	**.858**	.384	-.042	.811	.070	-.004	.239	***.292***	***.008***	***.576***
M is loyalty-betrayal, Y is seriousness of violence														
X = Persecutory delusions	.140	.019	-.140	-.395	.113	-.240	-.493	.012	***.099***	***.013***	***.265***	***.339***	***.083***	***.595***
X = Delusions involving doubles	.109	.048	.268	-.054	.592	.212	-.109	.534	.056	-.001	.185	.235	-.017	.488
M is fairness-injustice, Y is Cornell’s instrumental-reactive aggression														
X = Persecutory delusions	.259	.000	-.137	-.282	.008	-.062	-.199	.074	***-.074***	***-.172***	***-.013***	***-.263***	***-.406***	***-.121***
X = Somatic delusions	.253	.000	-.075	-.32	.177	.075	-.157	.308	***-.151***	***-.255***	***-.068***	***-.297***	***-.441***	***-.153***
X = Delusions of mind reading	.250	.000	-.043	-.302	.215	.052	-.179	.284	-.096	-.268	.063	***-.029***	***-.429***	***-.149***
X = Somatic/tactile hallucinations	.251	.000	-.042	-.289	.205	.055	-.165	.277	-.098	-.239	.019	***-.290***	***-.430***	***-.150***
M is loyalty-betrayal, Y is Cornell’s instrumental-reactive aggression														
X = Persecutory delusions	.249	.000	-.137	-.282	.008	-.063	-.201	.074	***-.073***	***-.170***	***-.015***	***-.250***	***-.389***	***-.110***
M is authority, Y is Cornell’s instrumental-reactive aggression														
X = Religious delusions	.096	.071	.041	-.098	.180	-.068	-.234	.097	***.109***	***.105***	***.243***	***.205***	***.024***	***.386***
X = Command hallucinations	.120	.036	***.164***	***.020***	***.308***	.115	-.045	.276	.048	-.018	.150	.186	-.054	.272

Bold: confidence interval does not move from positive to negative

Expert rated ‘loyalty-betrayal’ was the only moral cognition which was associated with homicide. Delusions of persecution were the only psychotic symptoms which correlated with ‘loyalty-betrayal’. The relationship between persecutory delusions and homicide was completely mediated by ‘loyalty-betrayal’. Although the overall correlation between persecutory delusions and homicide was negative (inverse), the mediation effect of ‘loyalty-betrayal’ revealed a positive relationship between persecutory delusions and homicide, provided ‘loyalty-betrayal’ was judged to be present by expert clinicians blind to the rating of delusions by treating clinicians. Within the mediation model, persecutory delusions that were not associated with ‘loyalty-betrayal’ were negatively associated with homicide, but persecutory delusions which were positively associated with ‘loyalty-betrayal’ were positively associated with homicide. ‘Loyalty-betrayal’ also had a direct effect on homicide when controlling for persecutory delusions. ‘Loyalty-betrayal’ did not mediate the relationship between delusions involving doubles and homicide, however, both made a significant contribution to the model.

Expert rated ‘loyalty-betrayal’ was also the only moral cognition that was associated with the seriousness of violence. The relationship between persecutory delusions and seriousness of violence was completely mediated and reversed by ‘loyalty-betrayal’. Similarly, for homicide, persecutory delusions that were not associated with ‘loyalty-betrayal’ were negatively associated with seriousness of violence, but persecutory delusions which were positively associated with loyalty-betrayal were also positively associated with seriousness of violence. ‘Loyalty-betrayal’ also had a direct effect on seriousness of violence when controlling for persecutory delusions.

Expert rated ‘fairness-injustice’ was negatively associated with Cornell’s instrumental-reactive aggression (i.e. positively associated with reactive violence). ‘Fairness-injustice’ completely mediated the relationship between persecutory delusions and instrumental violence, and partially mediated the relationship between somatic delusions and instrumental violence, making an independent contribution to the model in both cases.

Although ‘fairness-injustice’ was positively associated with somatic delusions and delusions of mind reading, it did not mediate the relationship between these psychotic phenomena and instrumental violence.

‘Loyalty-betrayal’ was also negatively associated with Cornell’s instrumental-reactive aggression scheme (i.e. positively associated with reactive violence). ‘Loyalty-betrayal’ completely mediated the relationship between persecutory delusions and instrumental violence, whilst making an independent contribution to the model.

Religious delusions and command hallucinations were both positively associated with moral cognitions involving ‘authority’. ‘Authority’ completely mediated the relationship between religious delusions and Cornell’s reactive-instrumental violence scheme, making an independent contribution to the model. In other words, religious delusions were positively associated with instrumental acts of violence when mediated through ‘authority’.

## Discussion

This is the first study investigating the role played by psychotic symptoms and moral cognitions at the time of the violent act, for forensic patients with schizophrenia or schizoaffective disorder. We sought to develop models to test if psychotic symptoms congruent with or in the form of moral cognitions could explain more that psychotic symptoms alone regarding a) their causal ‘relevance’ for serious acts of violence, b) the seriousness of violence, and c) the form of violence i.e. instrumental versus reactive.

### Key results summarised

Table 1 sets out a proposed model consisting of a series of hypothesised mediation pathways. These pathways occur between categories of delusion and hallucination modified from Peralta [54], categories of moral cognition [39] and forms and severity of violence based on our earlier findings of such links between moral dispositions and homicide in schizophrenia [12]. There was a significant mediated relationship between delusions of guilt and relevance to violence where mediation was via moral cognitions of care-harm. There was a significant mediated relationship between persecutory delusions and reactive violence, where mediation was via moral cognitions of fairness-injustice. There was a significant mediated relationship between persecutory delusions and reactive violence, where mediation was via moral cognitions of loyalty-betrayal. This finding was not in accordance with our original interpretation of Peralta’s classification but has face validity. We also found that delusions of persecution were related to the severity of violence and to homicide when mediated via moral cognitions of loyalty-betrayal in keeping with our earlier study of moral dispositions [12]. There was a direct effect of delusions of misidentification on seriousness of violence, though this was not mediated by any moral cognition. Religious delusions were related to instrumental violence when mediated via moral cognitions concerning authority. We did not find any relationship between Peralta’s classification of somatic and related delusions and hallucinations and moral cognitions concerning purity-disgust as we had expected.

Our findings suggest that moral cognition may be an important, but hitherto neglected, determinant of violence within this group. Within this cross-sectional sample, involving most of a national cohort (all of whom had a history of violence), expert witnesses identified moral cognitions as a motivating factor in the majority of cases. The most frequently identified moral cognitions concerned ‘fairness-injustice’ (63%), followed by ‘loyalty-betrayal’ (54.5%), ‘authority’ (30.9%), ‘purity-disgust’ (25.5%), and care-harm’ (20%). Of note, all five moral cognitions were evident within the sample.

Regarding our first hypothesis, the majority of psychotic symptoms present at the time of the violent act, or those judged to be ‘relevant’ to the violent act, were not positively associated with either the seriousness or the form of violence. Omnibus MANOVA tests showed significant associations between total psychopathology (SAPS total) and five moral cognitions, between form of violence (instrumental reactive aggression) and five moral cognitions, and between severity of violence and five moral cognitions. Eta^2^ statistics for each MANOVA ranged from 0.17 to 0.62 regarded as medium to large effect sizes.

In keeping with hypotheses 1 and 2, specific moral cognitions were associated with both the seriousness and form of violence even when controlling for neurocognitive impairment [48] or violence proneness [48, 65].

Regarding hypothesis 3, specific psychotic symptoms judged present or ‘relevant’ to the violent act were associated with specific moral cognitions in keeping with the model in Table 1 and where this did not hold true, other associations had face validity, where the explanation made sense (Table 6). Moral cognitions concerning ‘care-harm’ correlated positively with religious delusions, and grandiose delusions. Moral cognitions concerning ‘fairness-injustice’ correlated with persecutory delusions. Moral cognitions concerning ‘loyalty-betrayal’ also correlated with persecutory delusions. Moral cognitions concerning ‘authority’ correlated with religious delusions and command hallucinations. However, no delusion mapped in every case onto a specific moral cognition or vice versa – both forms of classification added explanatory power. There was a significant tendency for correlations between psychotic symptoms and moral cognitions to be stronger where the psychotic symptom was judged ‘relevant’ to the violent act. ‘Relevant’ symptoms were more likely to have a stronger association with moral cognitions than those symptoms that were merely present. Moreover, when psychotic symptoms were rated regarding their ‘relevance’ to violent acts, new relationships with face validity were observed. Moral cognitions concerning ‘care-harm’ were associated with ‘relevant’ delusions of guilt, and moral cognitions concerning ‘purity-disgust’ were associated with voices criticizing.

Although this study had a cross-sectional design, hypotheses 4 and 5 concerned the modelling of mediation relationships between independently rated psychotic symptoms, moral cognitions, and the seriousness and form of violence, using mediation analysis. For hypothesis 4, in some cases, the relationship between the presence of psychotic symptoms and their ‘relevance’ for acts of violence was statistically mediated by moral cognitions supporting a pathway from presence to ‘relevance’. For example, the relationship between persecutory delusions and their ‘relevance’ to a violent act was mediated by moral cognitions concerning ‘loyalty-betrayal’. Persecutory delusions become more ‘relevant’ for acts of violence when accompanied by thoughts concerning betrayal. In other cases, moral cognition contributed independently to the ‘relevance’ of symptoms for acts of violence when controlling for the presence of symptoms. For example, for a violent act, moral cognitions concerning ‘care-harm’ accounted for the ‘relevance’ of delusions of guilt even when controlling for the presence of delusions of guilt.

Hypothesis 5 concerned specific moral cognitions that mediated the relationship between specific psychotic symptoms and the seriousness or forms of violence (Table 7). For example, persecutory delusions were negatively related to homicide and the seriousness of violence within the overall sample, but when mediated via ‘loyalty-betrayal’ were positively associated with homicide and with the seriousness of violence. Delusions of persecution, in and of themselves, were not sufficient to account for the seriousness of violence, but within the context of feeling betrayed were more likely to lead to serious violence including homicide. Beliefs concerning being betrayed may, therefore, have particular significance for homicide and the seriousness of violence for patients with schizophrenia. In non-psychotic homicides, evidence from the forensic literature supports a relationship between intimacy and overkill [72]. In some cases, specific moral cognitions accounted for the form of violence even when controlling for the presence of psychotic symptoms. For example, moral cognitions concerning ‘authority’ were associated with instrumental violence, even when controlling for religious delusions. Religious delusions were only associated with instrumental violence when mediated via moral cognitions concerning ‘authority’. Patients who have religious delusions accompanied by moral cognitions concerning ‘authority’ may be particularly likely to carry out instrumental rather than reactive violence involving goal setting and planning. Currently, most structured risk assessment instruments do not distinguish between the risk of instrumental versus reactive violence.

Within this study, the majority of patients were experiencing persecutory delusions at the time of the violent act (74.5%) and the most common moral cognitions concerned ‘fairness-injustice’ (63.6%), followed by ‘loyalty-betrayal’ (54.5%), which may have contributed to our positive findings regarding their role as mediators. Fewer patients presented with delusions of jealousy (5.4%), which may explain the lack of association between these delusions and moral cognitions concerning ‘loyalty-betrayal’.

### Limitations

This study was cross-sectional and therefore the mediation models suggesting causal relationships should be treated cautiously. Correlation can never establish causation or the direction of causation. We have used mediation analysis to address this by examining for direct connections between specific delusions rated present or ‘relevant’ by treating clinicians. We controlled for specific moral cognitions judged to be present at the time of the act by using expert witnesses. The severity and form of violence were also rated independently. There are some patients for whom delusions do not have associated moral cognitions and there are some patients who may have had moral cognitions present at the time or ‘relevant’ to the violent act that were not related to delusions. It is unclear if this is a limitation of a small sample size. Ultimately, demonstrating a causal relationship between psychotic symptoms, moral cognition, and violence will require larger prospective studies with causal designs [10, 11]. We believe that the issue of whether moral cognitions are the key mediator between psychotic symptoms and acts of violence will only be solved via multiple replications and eventual meta-analyses.

The overriding value of this study is that it establishes a new paradigm for investigating violence carried out by patients with mental disorders. Not all violent acts are accounted for in this study. We have not included a category for ‘egoistic’ acts of violence that were not believed to be morally justified by the patient.

Similar to other studies in this area [11] we decided to forgo Bonferroni corrections because of the risk of type II error, which at this early stage would impede the development of new ways of conceptualizing determinants of violence and that may reduce catastrophic outcomes. We have discussed causal modelling in the light of mediation modelling only [67], although within the limits of what is appropriate in a scientific paper. We have carefully set out the limits of causal inference using Hayes process macro models for mediation analysis, again within the limits of what is appropriate in a research paper. By using mediation analysis, we have not sought to prove causation in every case, only that pathways are in keeping with the theoretical explanation in a significant proportion.

This study involved a small sample but one that consisted of the majority of a national forensic cohort of patients with schizophrenia or schizoaffective disorder.

Within the present study, only a small number of participants were female. Some forms of moral cognitions may be more significantly associated with homicide or violence amongst women. For example, moral cognitions concerning ‘care-harm’ may motivate filicide amongst females in keeping with research by Friedman et al., 2005 [13] and d’Orban’s concept of altruistic homicide [73].

This study was carried out in an Irish forensic psychiatric population, and the links between specific delusions, specific moral cognitions and seriousness and forms of violence may vary between cultures.

While there is currently no psychometrically sound measure of moral cognition for a specific act of violence, the moderate to good intraclass correlations observed between expert witnesses, admitting or treating clinicians, independent clinicians, and patients’ ratings of moral cognitions, support the validity of our approach. Also, because the explanation that moral cognitions in combination with psychotic symptoms could explain three discrete phenomena a) the relevance of symptoms for violent acts b) the seriousness of violence d) and whether the violence was reactive or instrumental, we believe this indicates construct validity.

The strengths of the study include the independence of ratings by expert witnesses and treating or admitting clinicians; the independence of the instrumental and reactive violence ratings; and the use of homicide as an objective discriminant, in addition to the sample consisting of most of a national forensic cohort. A further strength is the moderate to good intraclass correlations for moral cognitions between patient self-ratings and ratings by expert witnesses, treating or admitting clinicians and independent research clinicians.

### Interpretation

We have carefully sought out possible sources of bias in our approach to rating and analysis and sought to check for alternative interpretations. A delusion is generally taken as evidence of impaired information processing or impaired meta-cognitive reasoning and is often combined with anomalous experiences [53]. We have found in this sample of patients with schizophrenia or schizoaffective disorder who acted violently, that moral cognitions judged present were congruent with psychotic symptoms in a significant proportion of cases, though not all. We have shown there is a significant mediation relationship between a delusion and its ‘relevance’ for an act of violence via moral cognition (Tables 6). We have also shown that specific moral cognitions mediate the relationship between specific psychotic symptoms and the seriousness and form of violence outlined in Table 7, consistent with an earlier factor analysis [54]. Mediation accounts for large amounts of the variance of the dependent variable in some models.

We have previously shown that a measure of traits or dispositions regarding moral foundations distinguished between patients with schizophrenia or schizoaffective disorder who committed homicide when compared with similar patients who committed less serious acts of violence. Specifically, patients who endorsed more extreme dispositions towards loyalty and authority were more likely to have committed homicides [12]. In this study, we have shown that ‘loyalty’ as a state also related to seriousness of violence and homicide. Using the same conceptual framework as our previous exploratory study (Haidt’s five moral foundations, restated as domains of moral cognition) we have shown that ‘state’ moral cognition is related to the form of delusions relevant to violent acts and certain forms of violence (such as instrumentality and the severity of violence). Delusions of persecution were positively associated with homicide only when mediated by moral cognitions of loyalty-betrayal. We do not know if these patients also had ‘trait’ dispositions towards valuing ‘loyalty-betrayal’ over other ‘trait’ moral foundations as found in our earlier study. Such questions remain for future research. In any case, regardless of a person’s standing on each ‘trait’ moral foundation, in theory, it should be possible for them to experience moral cognitions associated with each discreet foundation depending on the situation that they find themselves in.

The relationship between an abnormal mood, such as fear or anger and violent acts has previously been described [18]. Similarly, the mediating role of anger between delusions and violent acts has been quantified [10, 11]. In this study, we have demonstrated a mediating role for moral cognitions between delusions, hallucinations, and qualities of violence. These findings raise the hypothesis for future consideration that delusions may be considered as manifestations of disturbed moral cognition, in the same way that delusions may be considered as manifestations of abnormal affects. Abnormal affects such as extreme, disproportionate or irrelevant fear or anger, impaired moral cognitive abilities, impaired reasoning (whether state or trait) and phenomena such as delusions and hallucinations may all be manifestations of the same psychotic processes such as impaired information processing [54] or abnormal neurophysiological function.

### Generalisability

Using a similar methodology, Coid et al. [11] observed that anger within the psychotic state has a mediating relationship between persecutory delusions and serious violence, a relationship that has explanatory power. This study suggests that moral cognitions have a mediating role between delusions and violence, and these findings may be compatible. Feelings of anger, anguish, and grief within the psychotic state may be strongly related to moral cognitions. For example, because of the powerful emotions associated with betrayal, the betrayed party may not act in their own best interest [74–76]. Individuals who believe they have been betrayed may discount potential consequences such as violent retaliation or almost certain imprisonment to act in accordance with their moral cognitions [32, 34–37, 74].

A scientific theory should have two properties [77, 78]: a) it has explanatory reach, and b) the explanation itself is ‘hard to vary’ where ‘hard to vary’ means not easily exchangeable with other explanations because the mechanism is functionally linked with the outcome and is constrained by existing knowledge. Within this paper, we have sought to apply theories from other domains of research to the study of schizophrenia [24]. A range of disciplines such as anthropology, history, and social psychology point to moral cognition as being a determinant of violence [24, 42, 43, 79]. This theory links the field of forensic psychiatry with the psychology of violence more generally.

We do not wish to confuse the empirical findings obtained within this single study with the assertion that the majority of psychotic patients who act violently do so for morally based reasons. In contrast, the findings obtained within this study simply corroborate aspects of the theory. All theories contain more than empirical findings [77].

Within this study, five domains of moral cognition derived from Haidt’s moral foundations’ theory could successfully be applied to the majority of cases, demonstrating the scope and reach of the theory. Moral cognition mediated between delusions and the seriousness and form of violence in some cases, though not all. For example, ‘loyalty-betrayal’ was associated with homicide and seriousness of violence, whereas ‘authority’ accounted for instrumental violence via mediation. In contrast, the majority of psychotic symptoms judged to be present or ‘relevant’ to a violent act were not positively associated with either the seriousness or form of violence.

The theory that moral cognition is a key determinant of violence for patients with schizophrenia is ‘hard to vary’. The abstract features of moral cognition, universal, justified, actionable and punishable, are functionally relevant to violent acts [24–33]. Not only are the theory and the findings of this study compatible with existing beliefs embodied within legal principles such as the M’Naughton rules, and epidemiological studies demonstrating an association between psychotic symptoms and violence [15], but the theory also appears to solve two important paradoxes within the field of forensic mental health. First, despite patients [80], clinicians, and the courts believing that psychotic symptoms are a determinant of violence [6], and despite epidemiological evidence supporting an association [1, 2] it has been difficult to empirically demonstrate a causal relationship [8, 9]. The theory that moral cognition is the mediating link between symptoms and violence offers a causal explanation.

Patients experiencing psychotic symptoms, such as delusions and hallucinations, may carry out a violent act when they are morally motivated to do so. When they subjectively believe that acting violently is the ‘right thing’ to do. These ‘morally’ motivated acts may include the following. A patient may decide to act violently with the intention to kill because they believe an individual (e.g. their significant other) will suffer intolerably unless they act (‘care-harm’); they may be angry over perceived persecution at the hands of an external agency (e.g. the police) and decide to take justice into their own hands (‘fairness-injustice’); they may act violently while experiencing anger or grief, believing they have been betrayed by someone close to them such as a family member (‘loyalty-betrayal’); they may act violently whilst in awe of and acting in accordance with a higher power such as God or the devil (‘authority’); they may believe a person to be impure or disgusting and thus deserving of violent punishment (‘purity-disgust’).

The second paradox resolved by the theory is that despite patients, clinicians, and the courts believing that psychotic symptoms may be the key determinant of violence, it has been observed that the homicide rate amongst patients with schizophrenia tracks changes in the homicide rate within the general population [4, 81, 82], calling into question the role played by symptoms alone. The theory that moral cognition is a determinant of violence may resolve this paradox and explain the association between the homicide rate amongst the mentally ill and the general population. Culture and personal dispositions may, therefore, determine the properties of an individual’s moral cognitions during specific non-independent ‘life events’ [11, 83]. Patients who experience psychotic symptoms, such as persecutory delusions, who come from cultures or subcultures which legitimize violence or extreme self-defence in response to potential threat may choose ‘fight’ rather than ‘flight’; gender-based violence may also be culturally mediated and related to ‘honour’ [83, 84]. Within our study, many of the acts of violence were judged to be reactive (69%), rather than instrumental or offensive aggression (30%) and primarily concerned injustice and betrayal, which may differ within other cultures or populations. Moral cognition represents a unifying construct for many forms of violence including gender-based violence [85], cultural differences [86, 87] and aspects of psychopathology other than psychosis [86]. Recent research on the specific topic of gender-based violence has found that men convicted of domestic violence have a high moral self-concept and a sacred vision of the five moral foundations [85]. The potential resolution of these two paradoxes as outlined above may be regarded as a strength of the theory that moral cognition is an important mediator between psychotic symptoms and acts of violence.

While there is no history of naming moral delusions or moral paranoia that we are aware of, there is a distinction between the structure and dynamics of psychopathological phenomena. Patients’ moral justification for carrying out a violent act may be best conceptualised as a metacognitive error rather than the delusion or hallucination in and of itself. A delusion might shift the moral ‘structure’ to form a set of beliefs, a structural change that governs the person’s actions. Misinterpretations and confrontations might give rise to dynamic changes such as emotions, feelings and reactions. Both structural and dynamic changes may lead to aggressive reactions which are based on the psychopathology of the patient with delusions [88, 89] and this may be relevant to risk assessment and treatment.

Specific delusions and specific moral cognitions correlate significantly but do not perfectly coincide. Because not all delusions will evoke a moral cognition, and since not all delusions of the same type will evoke the same moral cognition, the demonstration that specific delusions are relevant to the form and severity of violence when mediated by specific moral cognitions is the point of our paper. This is much the same point made by Coid’s group [10, 11] regarding the mediating role of anger for delusions leading to violence. Not all delusions lead to anger, but those that do are more closely related to violence [18, 19]. Delusions appear to be necessary but not sufficient in these models.

If the concept of ‘delusion’ is expanded repeatedly to accommodate new findings such as moral cognition or anger, the paradigm of ‘delusion’ may become unscientific. In the alternative, ‘angry’ and ‘moral’ may be inherent but new aspects of ‘delusion’ which are not accommodated within current definitions or concepts. If moral cognitions are accepted as a mediating factor between delusions and forms and severity of violence, it may be possible to develop a new generation of structured professional judgement tools for risk and needs assessment involving delusions and hallucinations, their associated moral cognition and the related affects. Such a scheme is consistent with findings demonstrating a relationship between delusions of persecution, anger and serious violence [10, 11]. One limitation facing current violence risk assessment tools is the ‘spectre of randomness’, or the unpredictability of ‘life events’, which trigger acts of violence [90, 91]. However, many of these apparently ‘independent life events’ may not be truly random and may arise in part out of an individual’s genetic code, personality traits, moral dispositions, and moral cognitions i.e. ‘non-independent life events’ [90].

Separate to violence risk assessment, it may also be possible to develop new treatment programs for reducing violence recidivism. To date, these programs have focused on altering non-causal risk factors [92, 93]. However, if patients lose contact with reality and believe that they are acting righteously, they may feel compelled or obliged to do so again. Psychological therapies, such as metacognitive training, may be directed towards teaching patients not to jump to moral conclusions, while cognitive behavioural therapy might involve challenging the thinking underpinning moral beliefs or categorizations.

### Future research

The overarching theory that moral cognition explains a large proportion of violence carried out by patients with delusions and hallucinations generates many testable hypotheses. We have examined some hypotheses in this paper, however, much more needs to be done. Future studies will necessitate the development of new instruments for reliably measuring patients’ moral cognition. Demonstrating a causal relationship between moral cognition and violence will be difficult. However, in principle delusions can be distinguished from moral cognitions. Delusions are held to be fixed, false, and unshakable beliefs that cannot be changed by evidence and are not culturally explained [53]. In contrast, moral cognitions possess certain fundamental abstract properties: they are universal (i.e. independent of local laws or customs), justified (the right thing), actionable (i.e. that the moral cognition demands action), and moral infringements are punishable or may be used to legitimise the use of force (which may include violence). Patients tormented by abstract entities and who believe they are ‘unjustly’ treated may be unable to act on their delusions because of the absence of a person to punish. One study involving 70 patients with persecutory delusions found that 16% involved paranormal persecutors including God, the devil, aliens, spirits, witches and wizards [94].

Before conducting a prospective study to robustly test for causation it would be instructive to compare violent and non-violent patients using a case-control design regarding their psychotic symptoms and their degree of preoccupation with moral cognition, to determine if moral cognitions and the specific dimensions of moral thought discriminate between violent and nonviolent groups. Should moral cognitions prove to be an effective discriminant between violent and nonviolent patients with schizophrenia a large scale prospective study could be conducted to corroborate the mediating role played by moral cognitions. The findings from the current study may facilitate power analysis for these future studies.

In addition to clarifying the role played by moral cognitions by patients with psychotic disorders, future research should also explore the role played by moral cognition and acts of violence for those with personality traits or constellations of traits associated with psychopathic, antisocial, narcissistic, and borderline personality disorders. As a diagnosis of a personality disorder is neither necessary nor sufficient for serious violence, such as homicide or serial killing [95, 96], moral cognition may also be an important mediator within these groups. One study has shown that within the U.S., the states that placed greater importance on social standing regarding one’s person, family, reputation, and property, had more than twice as many school shootings per-capita as those that did not [84]. Since behavioral scientists are people first and scientists second, it is difficult to empathise with the perpetrator, and easy to solely attribute their acts to personality traits (e.g. paranoid, narcissistic, sadistic, or psychopathy), rather than considering possible moral motivations (e.g. a response to a betrayal or perceived injustice) [97]. A focus on moral cognition within these groups may, also have explanatory value informing risk assessment and treatment programmes.

Although the majority of violence may be morally motivated, it is likely that some individuals will act violently solely for ‘egoistic or selfish’, rather than morally based reasons (e.g. bank robbers). The distinction between ‘egoistic’ and morally motivated violence may mirror the dichotomy between mental systems or adaptations focusing on immediate short-term gain (i.e. over-optimistic future discounting) [98, 99], versus those focusing on long term wellbeing [100, 101]. Also, recent research points to moral or justified violence and ‘egoistic’ or unjustified violence being subserved by different neural systems [102]. Many criminals, such as sex-offenders, who carry out a violent act, present with cognitive distortions. However, it remains unclear to what degree they believe these rationalisations themselves [103, 104]. It is conceivable that an individual carries out an act of violence for selfish reasons, and deploys self-deceiving cognitive distortions, not only to deceive the ‘self’ but to better deceive others [105, 106]. Moral cognitions are thought to be the product of adaptive mechanisms for facilitating communal living [107], a proxy for survival and reproduction, whereas ‘egoistic’ cognition and violence are more directly connected to these Darwinian goals [34, 108]. Self-deception regarding moral motivations may achieve both distal and proximal objectives.

## Conclusions

The psychopathological understanding of structural and dynamic change experienced by patients with schizophrenia is part of a long discourse within psychiatry [88, 89]. Our findings suggest that moral cognition appears to be relevant for acts of violence carried out by patients with schizophrenia or schizoaffective disorder. The theory that moral cognition is a key determinant of violence also solves important paradoxes within the field and may lead to the development of a new generation of violence risk and needs assessment instruments [109]. This theory may also lead to new prevention and intervention programs. Our findings are consistent with a broad movement within psychology and anthropology exploring violence and morality [24]. The findings are also consistent with the M’Naughten rules which for the past two hundred years have acted as a bulwark protecting those who acted under the influence of mental illness from harsh consequences. We acknowledge that not all violence is moral (communal) in nature and that behaviour can also be ‘egoistic’ (selfish). The distinction between ‘egoistic’ and moralistic violence may be in and of itself instructive, and when combined with the instrumental reactive dichotomy, provide a unified theory of violence. This inference echoes the work of Charles Darwin’s Origin of the Species: “In the distant future, I see open fields for far more important researches. Psychology will be based on a new foundation, that of the necessary acquirement of each mental power and capacity by gradation” [110]. Egoistic violence may come first, with moralistic violence being a later development. Both forms of violence may require different approaches to prediction, management, and treatment. We suggest that the distinction between moralistic and egoistic violence may also help to decrease stigma experienced by a minority of patients who carry out acts of violence when unwell. There is now an urgent need to carry out a range of studies investigating moral cognition and violence amongst patients with schizophrenia.

## Data Availability

The dataset used and/or analysed during the current study are available from the corresponding author on reasonable request.

## Abbreviations

CMH: Central Mental Hospital
DUNDRUM-1: Dangerousness, UNDerstanding, Recovery, Urgency, Manual triage security scale
HCR-20: The Historical Clinical Risk management-20
ICC: Interclass correlations
MCCB: MATRICS consensus cognitive battery
NFMHS: National forensic mental health service
NGRI: Not guilty by reason of insanity
OR: Odds ratio
SAPS: Schedule for the assessment of psychotic symptoms
SCID-I: Structured clinical interview for diagnostic and statistical manual 4th edition
